# Two-Dimensional Electromagnetohydrodynamic (EMHD) Flows of Fractional Viscoelastic Fluids with Electrokinetic Effects

**DOI:** 10.3390/nano12193335

**Published:** 2022-09-25

**Authors:** Kai Tian, Shujuan An, Guangpu Zhao, Zhaodong Ding

**Affiliations:** 1 School of Mathematical Science, Inner Mongolia University, Hohhot 010021, China; 2 College of Sciences, Inner Mongolia University of Technology, Hohhot 010051, China

**Keywords:** electromagnetohydrodynamic (EMHD), fractional Maxwell model, electrokinetic effect, slip length, resonance behavior

## Abstract

The present study provides analytical and numerical solutions for an electromagnetohydrodynamic (EMHD) flow using a Caputo time-fractional Maxwell model. The flow is a typical rectangular channel flow. When the scale of the cross-stream is much smaller than the streamwise and spanwise scales, the model is approximated as a two-dimensional slit parallel plate flow. Moreover, the influence of the electric double layer (EDL) at the solid–liquid interface is also considered. The electro-osmotic force generated by the interaction between the electric field and the EDL will induce a flow (i.e., electro-osmotic flow). Due to the application of the electric field at the streamwise and the vertical magnetic field, the flow is driven by Lorentz force along the spanwise direction. Simultaneously, under the action of the magnetic field, the electro-osmotic flow induces a reverse Lorentz force, which inhibits the electro-osmotic flow. The result shows that resonance behavior can be found in both directions in which the flow is generated. However, compared with the classical Maxwell fluid, the slip velocity and resonance behavior of fractional Maxwell fluid are suppressed. In the spanwise direction, increasing the strength of magnetic field first promotes the slip velocity and resonance behavior, and then suppresses them, while in the streamwise direction, both the electro-osmotic flow and resonance behavior are suppressed with the magnetic field.

## 1. Introduction

Microfluidic devices are greatly utilized in many fields, such as biological engineering, the transport of chemicals in the body and heat transfer in electronic components [1,2]. Microfluidic transport can be realized by pressure-driven micropumps [3,4], electro-osmotic micropumps [5,6,7,8], and electromagnetohydrodynamic (EMHD) pumps [9,10,11]. The EMHD micropump is driven by the Lorenz force, which is produced by the interaction of an external electric field and a magnetic field [12]. Compared with other types of micropumps, the EMHD micropump has several advantages, namely, a simple manufacturing process, continuous flow power and two-way pumping capacity. For instance, it can be used in fluid pumping, flow control in fluidic networks and fluid stirring and mixing [10]. In addition, the EMHD micropump can reduce energy consumption and save costs in industry, so related areas of study have always attracted attention from researchers, such as rotating EMHD pumps [11], stagnation point flow [13], EMHD flow in corrugated walls [14], etc. Recently, Khan et al. [15,16] considered the impact of gyrotactic microorganisms on the nonlinear mixed convective MHD flow of thixotropic and Prandtl–Eyring nanofluids. They examined the variations of heat transfer characteristics subject to nonlinear radiative flux and heat source/sink, and found that for larger thixotropic fluid parameters, the velocity field boosts up, while for rising values of the Hartmann number, the velocity and temperature have opposite behaviors. Furthermore, at the channel walls, most solids spontaneously acquire surface electric charges when brought into contact with a polar medium, and then the number of positive and negative ions in the solution near the solid–liquid interface is inconsistent, forming an electric double layer (EDL). The electro-osmotic force generated by the interaction between the electric field and the EDL will move the fluid along the electric field. In EMHD flow, however, the influence of the electro-osmotic force is often ignored (such as Refs. [10,11,12,13,14,17,18]). Therefore, the coupling effect between EMHD flow and electro-osmotic flow needs to be further investigated, which is one of the intentions of this paper.

Viscoelastic fluids (e.g., blood and polymer solutions) are often used in microfluidic transports. Since the fluids with both elastic and viscous properties, in the flow field, deform/flow under external force, the external force is vanished, and the deformation will return to the specified threshold with time evolution [19]. Due to this characteristic, a viscoelastic fluid often experiences resonance during the flow process, i.e., it increases abruptly the oscillation amplitude of a system when an external force matches the system’s natural frequency [20]. The resonance behavior with viscoelastic fluid flow has been studied by many researchers. Yin and Zhu [21] studied the unidirectional oscillating flow produced by a periodic pressure gradient through the fractional Maxwell model. Andrienko et al. [22] investigated resonance-like phenomena in axisymmetric Poiseuille flows of viscoelastic fluids. Lambert et al. [23] analyzed the heat transfer enhancement in an oscillatory flow of a viscoelastic fluid in tubes. Tsiklauri and Beresnev [24] found sharp enhancements of the flow through researching the process of transition from a dissipative regime to an elastic regime with Maxwell fluids. Emilio Herrera [25] predicted flow enhancement on the pulsating flow in an aqueous worm-like micellar solution of cetyltrimethyl ammonium tosilate for various concentrations. In two-dimensional EMHD flow, will the viscoelastic fluid also have a similar flow enhancement phenomenon? Another purpose of this paper is to describe the resonance behavior during flow using a viscoelastic model.

In recent years, fractional calculus has been widely used in the research of abnormal diffusion, viscoelastic model, and soft materials [21]. Compared with the integer calculus, fractional calculus can more concisely and accurately describe the physical process with historical memory and spatial correlation [26]. The germination of fractional calculus can be traced back to L’ Hospital’s letter to Leibniz in 1695. After a series of work of many researchers, including Fourier, Abel, Liouville, and Caputo [27], the embryonic form of fractional calculus came out. Nowadays, fractional calculus has developed into a systematic branch of mathematics, and it is widely used in various fields [28]. Feng et al. [29] used the time-distributed Caputo fractional model to study the rotating electro-osmotic slip flow of generalized Maxwell fluids in a periodic electric field. Cao et al. [30] numerically investigated the electro-osmotic flow of double layers consisting of Newtonian fluid and fractional second-order fluid with a rotating frame in a parallel microchannel. Yang et al. [31] studied the electro-osmotic flow of Maxwell fluids with Riemann–Liouville fractional derivatives in a rectangular microchannel, and proposed the nonlinear conjugate gradient method to obtain the viscoelastic parameters. Abdulhameed et al. [32] explored unsteady pressure-driven and EMHD flow of an incompressible Maxwell fluid with time-fractional Caputo–Fabrizio derivatives through a circular tube. Abro and Solangi [33] analytically researched the heat transfer and free convection problem in second-grade fluid with porous impacts using Caputo–Fabrizoi fractional derivatives. Liu et al. [34] studied the unsteady EMHD flow of fractional Oldroyd-B fluids between parallel plates on heat transfer. Inspired by these studies, the fractional Maxwell model was chosen to explore the characteristics of viscoelastic fluid flow.

Furthermore, the velocity slip on the channel wall is also an important feature in micro/nano-scale flow [35,36,37]. In macro-fluid flow, the no-slip boundary condition is applied successfully to model some experiments. This success may not always reflect the accuracy of the boundary condition but may reflect the insensitivity of the experiment to the boundary condition [38]. At the microscale level, slip boundary conditions will become important when the length scale over which the liquid velocity changes approaches the slip length in a channel [39], where the slip length is the distance inside the walls at which the fluid velocity extrapolates to zero. Pascall and Squires [40] measured the induced charge of electro-osmosis over gold electrodes; both the magnitude and frequency dependence of the measured slip velocity are captured quantitatively by accounting for the physical capacitance and surface chemistry of the dielectric layer. Galea and Attard [41] presented a model of atomic roughness to study the effect of solid roughness on the slip boundary condition during shear flow. By using evanescent waves, Bouzigues [42] measured velocity profiles and diffusion profiles in pressure-driven and electro-osmotic flows, and determine the hydrodynamic slip lengths with 10 nm accuracy in the Debye layer for hydrophilic and hydrophobic surfaces. Khair and Squires [43] found that an enhancement occurs in the electrophoretic motion of a colloidal particle whose surface exhibits hydrodynamic slip.

As already mentioned, viscoelastic fluids have resonance phenomena in a tube/channel flow. Whether the resonance behavior occurs at the micro-nano scale is exactly what we want to know. At the micro-nano scale, electrostatic force is particularly important, so on the basis of previous research [1,12,17,44], we extended the research of Jian [44] to fractional viscoelastic fluids to study the resonance behavior in channel flow. For this purpose, current paper investigates the EMHD flow of fractional viscoelastic fluids with electro-osmosis and velocity slip effects. The special feature of this model is that the Lorentz force has a component in the direction of the electric field. The scale of the cross-stream (height) is much smaller than the streamwise (length) and spanwise (width) scales (i.e., approximately an infinite slit plate). The physical mechanism is as follows. Due to the application of the electric field at the streamwise direction and the vertical magnetic field, the flow is driven by Lorentz force, which originates from the interaction between an imposed periodic electric field and the magnetic field, and its direction is along the spanwise direction. Moreover, the influence of the electric double layer (EDL) at the solid–liquid interface is also considered. The electro-osmotic force generated by the interaction between the electric field and the EDL will also induce a flow (i.e., electro-osmotic flow). Therefore, for such a two-dimensional channel flow, the Fourier transform can be used to obtain an analytical solution. Meanwhile, a numerical algorithm can also be obtained by the finite difference method. The effects of several dimensionless numbers, such as the Hartmann number Ha, fractional parameters α, and slip length *L*, on the velocity and resonance behavior are analyzed graphically in detail. The result shows that resonance behavior can be found in both directions in which the flow is generated. However, compared with the classical Maxwell fluid, the slip velocity and resonance behavior of fractional Maxwell fluid are suppressed. In the spanwise direction, increasing the strength of magnetic field first promotes the slip velocity and resonance behavior, and then suppresses them. In the direction of the electric field, both electro-osmotic flow and resonance behavior are suppressed with the magnetic field. The rest of this paper is arranged as follows. In Section 2, the problem is formulated, and solutions of the EMHD velocity are presented. In Section 2.1, the net charge density is given by solving the linearized Poisson–Boltzmann equation. The Caputo fractional derivative and the governing equation are presented in Section 2.2. In Section 2.3, the Fourier transform method is used to obtain velocity expression. A finite difference scheme is provided for the velocity distribution in Section 2.4. Velocity distribution and the resonance of fractional viscoelastic fluid are investigated in Section 3.1 and Section 3.2, respectively. Finally, the study is summarized and concluded in Section 4.

## 2. Mathematical Modeling

**Figure 1:**
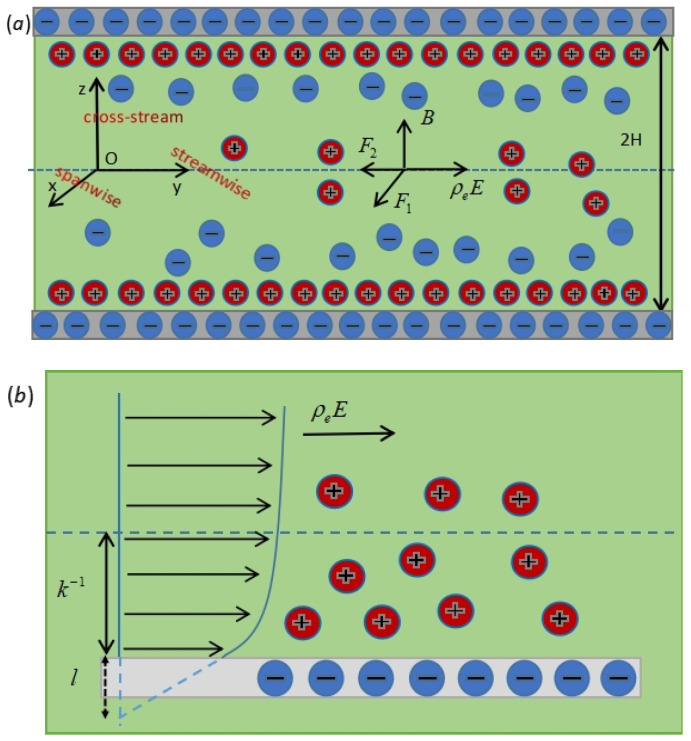
(**a**) Sketch of EMHD flow with EDL effect in parallel microchannels. (**b**) Sketch of the slip length at the wall, where *l* is the distance inside the wall where the fluid velocity would extrapolate to zero and $k^{-1}$ is the thickness of the electric double layer.

In this paper, we studied the slip flow of EMHD pump combined with the EDL effect in the micro-parallel channel. The model sketch is shown in Figure 1. The electrical field E(t) and a steady magnetic field of strength *B* are applied between two negatively charged micro-parallel plates separated by a distance 2H in the *y*-axis (streamwise) and *z*-axis (cross-stream) directions, respectively. The flow is a typical rectangular channel flow, as indicated in Section 1, assuming that the scale of the cross-stream is much smaller than the streamwise and spanwise scales. In this case, the model can be approximated as a two-dimensional slit-parallel plate flow. It can be seen from Figure 1a that the forces acting on the fluid during the entire flow are only electro-osmotic force and Lorentz force, and the velocity in the *x*-axis and *y*-axis directions are functions of *z* and *t*. Thus, the velocity vector can be expressed as $U=\left(u(z,t),v(z,t),0\right)$. The electro-osmotic force acting on the fluid is $\rho_{e}E$ along the *y*-axis direction. The Lorentz force $F=J\times B=\sigma B\left(E-Bu,-Bv,0\right)$ has two components, one of which is perpendicular to the electric and magnetic fields, and the other is parallel to the direction of the electric field, where the local ion current density is $J=\sigma \left(E+U\times B\right)=\sigma \left(Bv,E-Bu,0\right)$. For convenience, we shall denote $F_{1}=\sigma B\left(E-Bu\right)$ and $F_{2}=-\sigma B^{2}v$, respectively.

For the walls at z=±H, the slip boundary condition will be used. The so-called slip boundary condition means that on the solid wall (on the boundary), the velocity of the fluid is different from that of the solid wall, that is, the slip velocity is generated (see Figure 1b). Here, the partial slip boundary condition is used, which means that the tangential velocity of the boundary has a certain gradient. Navier pointed out that the velocity of the fluid at the solid wall is proportional to the gradient of the fluid velocity along the normal to the boundary surface, where the proportionality factor is called the slip length. The slip length *l* is the distance inside the wall, where the fluid velocity would extrapolate to zero [45].

### 2.1. The Local Net Charge Density and the Electrical Potential

The local net charge density $\rho_{e}$ and the electrical potential ψ(z) are described by the following Poisson–Boltzmann equations [19,46]:

(1) $$\begin{array}{cc} \; & \nabla^{2}\psi \left(z\right)=-\frac{\rho_{e}\left(z\right)}{\epsilon },\\ \; & \rho_{e}\left(z\right)=z_{\nu }e\left(n_{+}-n_{-}\right)=-2n_{0}z_{\nu }e\sinh\left[\left(z_{\nu }e\psi \right)/\left(k_{b}T\right)\right], \end{array}$$

where $n_{\pm }=n_{0}\exp\left[\mp \left(ez_{\nu }\psi \right)/\left(k_{b}T\right)\right]$ are the ionic number concentrations for the cations and anions in the liquid, respectively, ϵ is the dielectric coefficient of the electrolyte liquid, *e* is the electron charge, $z_{\nu }$ is the valence, $k_{b}$ is the Boltzmann constant, $n_{0}$ is the ion density of bulk liquid, and *T* is the absolute temperature. When the potential ψ(z) is low, the Debye–Hückel approximation can be applied to obtain the linearized equation [19,46]

(2) $$\frac{d^{2}\psi }{dz^{2}}=k^{2}\psi ,\;\mathrm{with}\;k^{2}=\frac{2n_{0}z_{\nu }^{2}e^{2}}{\epsilon k_{B}T}.$$

The boundary conditions of potential are given as follows:

(3) $$\psi =\psi_{0},\;\mathrm{at}\;z=\pm H,$$

where $\psi_{0}$ is the potential at walls. The solution of Equation (2) subject to the condition Equation (3) is given by

(4) $$\psi \left(z\right)=\psi_{0}\frac{\cosh(kz)}{\cosh(kh)},\;-H\leq z\leq H.$$

Using the above potential distribution, the net charge density can be expressed as

(5) $$\rho_{e}\left(z\right)=-\epsilon k^{2}\psi_{0}\frac{\cosh(kz)}{\cosh(kH)}.$$

### 2.2. Caputo Fractional Derivative and the Governing Equation

The Caputo fractional derivative is defined as [29]

(6) $$D_{t}^{p}f\left(t\right)=\left\{\begin{array}{cc} \frac{1}{\Gamma (n-p)}\int_{0}^{t}\frac{f^{\left(n\right)}\left(s\right)}{{\left(t-s\right)}^{p-n+1}}ds, & n-1<p<n,\;\\ \frac{d^{n}f(t)}{dt^{n}}, & p=n\in N, \end{array}\right.$$

where *p* is the fractional derivative parameter, and Γ(·) is the Gamma function.

The Navier–Stokes equation is

(7) $$\rho \frac{\partial u}{\partial t}+\rho \left(u\cdot \nabla \right)u=-\nabla P+\nabla \cdot \tau +f,$$

where ρ is the fluid density, *P* is the pressure, τ is the stress tensor, and f is the external body force vector.

The fractional Maxwell constitutive equation is given by

(8) $$\left(1+\lambda^{\alpha }D_{t}^{\alpha }\right)\tau =\mu \lambda^{\beta -1}D_{t}^{\beta -1}\dot{\gamma },\;\mathrm{with}\;0\leq \alpha \leq \beta \leq 1,$$

where $\dot{\gamma }$ is the shear rate, λ is the relaxation time of the fluid, μ is a dynamic viscosity, and α and β are the fractional derivative parameters. When α=β=1, it reduces to the ordinary Maxwell model, while for α=0 and β=1, it is simplified as the classical Newtonian fluid [47].

In the case of low Reynolds number flow, the inertia term can be ignored. Based on the assumption that the pressure gradient between the plates is zero, the simplified governing equations are obtained:

(9) $$\left\{\begin{array}{c} \begin{array}{c} \rho \frac{\partial u}{\partial t}=\frac{\partial \tau_{zx}}{\partial z}+\sigma B\left(E-Bu\right),\\ \rho \frac{\partial v}{\partial t}=\frac{\partial \tau_{zy}}{\partial z}+\rho_{e}E-\sigma B^{2}v. \end{array} \end{array}\right.$$

The simplified constitutive equation is

(10) $$\left\{\begin{array}{c} \begin{array}{c} \left(1+\lambda^{\alpha }D_{t}^{\alpha }\right)\tau_{zx}=\mu \lambda^{\beta -1}D_{t}^{\beta -1}\left(\frac{\partial u}{\partial z}\right),\\ \left(1+\lambda^{\alpha }D_{t}^{\alpha }\right)\tau_{zy}=\mu \lambda^{\beta -1}D_{t}^{\beta -1}\left(\frac{\partial v}{\partial z}\right). \end{array} \end{array}\right.$$

Substituting Equation (9) into Equation (10), we obtain

(11) $$\left\{\begin{array}{c} \begin{array}{c} \left(1+\lambda^{\alpha }D_{t}^{\alpha }\right)\left[\rho \frac{\partial u}{\partial t}-\sigma B\left(E-Bu\right)\right]=\mu \lambda^{\beta -1}D_{t}^{\beta -1}\left(\frac{\partial^{2}u}{\partial z^{2}}\right),\\ \left(1+\lambda^{\alpha }D_{t}^{\alpha }\right)\left[\rho \frac{\partial v}{\partial t}-\left(\rho_{e}E-\sigma B^{2}v\right)\right]=\mu \lambda^{\beta -1}D_{t}^{\beta -1}\left(\frac{\partial^{2}v}{\partial z^{2}}\right). \end{array} \end{array}\right.$$

The slip boundary conditions are

(12) $$\left\{\begin{array}{c} \begin{array}{c} {\left[u\pm l\frac{\partial u}{\partial z}\right]}_{z=\pm 1}=0,\\ {\left[v\pm l\frac{\partial v}{\partial z}\right]}_{z=\pm 1}=0. \end{array} \end{array}\right.$$

Introduce a set of dimensionless parameters as follows:

(13) $$\begin{array}{cc} \; & \bar{z}=\frac{z}{H},\;\bar{u}=\frac{u}{U_{eo}},\;\bar{v}=\frac{v}{U_{eo}},\;K=kH,\;\bar{t}=\frac{t\mu }{\rho H^{2}},\;L=\frac{l}{H},\;G=\sqrt{\frac{\sigma }{\mu }}\frac{E_{0}H}{U_{eo}},\\ \; & H\;a=BH\sqrt{\frac{\sigma }{\mu }},\;De=\frac{\lambda \mu }{\rho H^{2}},\;\bar{E}=\frac{E}{E_{0}}\;\mathrm{with}\;U_{eo}=-\frac{\epsilon E_{0}\psi_{0}}{\mu }, \end{array}$$

where $E_{0}$ is the maximum value of the electric field *E*. The dimensionless forms of Equations (11) and (12) are as follows:

(14) $$\left\{\begin{array}{c} \begin{array}{c} \left(1+De^{\alpha }D_{\bar{t}}^{\alpha }\right)\left\{\frac{\partial \bar{u}}{\partial \bar{t}}-\left[H\;aG\bar{E}-H\;a^{2}\bar{u}\right]\right\}=De^{\beta -1}D_{\bar{t}}^{\beta -1}\left(\frac{\partial^{2}\bar{u}}{\partial {\bar{z}}^{2}}\right),\\ \left(1+De^{\alpha }D_{\bar{t}}^{\alpha }\right)\left\{\frac{\partial \bar{v}}{\partial \bar{t}}-\left[K^{2}\frac{\cosh\left(K\bar{z}\right)}{\cosh\left(K\right)}\bar{E}-H\;a^{2}\bar{v}\right]\right\}=De^{\beta -1}D_{t}^{\beta -1}\left(\frac{\partial^{2}\bar{v}}{\partial {\bar{z}}^{2}}\right), \end{array} \end{array}\right.$$

(15) $$\left\{\begin{array}{c} \begin{array}{c} {\left[\tilde{u}\pm L\frac{\partial \tilde{u}}{\partial \bar{z}}\right]}_{\bar{z}=\pm 1}=0,\\ {\left[\tilde{v}\pm L\frac{\partial \tilde{v}}{\partial \bar{z}}\right]}_{\bar{z}=\pm 1}=0. \end{array} \end{array}\right.$$

### 2.3. Analytical Solutions of the EMHD Velocity Field

In order to obtain the analytical solution, we introduce the Fourier transform

(16) $$\tilde{X}\left(s\right)=\int_{-\infty }^{+\infty }X\left(t\right)e^{-ist}\mathrm{dt},$$

where *s* is a real parameter. It should be mentioned here that the use of Fourier transform or Laplace transform to solve fractional differential equations is very mature. The analytical solution obtained by using the method of similarity transformation is currently the most common method for dealing with fractional differential equations [48]. Then, using the Fourier transform with respect to $\bar{t}$ for the Equation (14), we obtain the following equation:

(17) $$\left\{\begin{array}{c} \begin{array}{c} \left[1+{\left(is\right)}^{\alpha }De^{\alpha }\right]\left(is\tilde{u}-H\;aG\tilde{E}+H\;a^{2}\tilde{u}\right)={\left(is\right)}^{\beta -1}De^{\beta -1}\frac{d^{2}\tilde{u}}{d{\bar{z}}^{2}},\\ \left[1+{\left(is\right)}^{\alpha }De^{\alpha }\right]\left(is\tilde{v}-K^{2}\frac{\cosh\left(K\bar{z}\right)}{\cosh\left(K\right)}\tilde{E}+H\;a^{2}\tilde{v}\right)={\left(is\right)}^{\beta -1}De^{\beta -1}\frac{d^{2}\tilde{v}}{d{\bar{z}}^{2}}, \end{array} \end{array}\right.$$

The simplified form of Equation (17) is

(18) $$\left\{\begin{array}{c} \begin{array}{c} \frac{d^{2}\tilde{u}}{d{\bar{z}}^{2}}-A\tilde{u}=-BH\;aG,\\ \frac{d^{2}\tilde{u}}{d{\bar{z}}^{2}}-A\tilde{v}=-BK^{2}\frac{\cosh\left(K\bar{z}\right)}{\cosh\left(K\right)}, \end{array} \end{array}\right.$$

with the boundary conditions

(19) $$\left\{\begin{array}{c} \begin{array}{c} A=\frac{1+{\left(isDe\right)}^{\alpha }}{{\left(isDe\right)}^{\beta -1}}\left(is+H\;a^{2}\right),\\ B=\frac{1+{\left(isDe\right)}^{\alpha }}{{\left(isDe\right)}^{\beta -1}}\tilde{E}. \end{array} \end{array}\right.$$

By solving Equation (18), the following equations are obtained:

(20) $$\left\{\begin{array}{c} \begin{array}{c} \tilde{u}=\frac{BH\;aG}{A}\left[1-\frac{\cosh\left(\sqrt{A}\bar{z}\right)}{\cosh\sqrt{A}+L\sqrt{A}\sinh\sqrt{A}}\right],\\ \tilde{v}=\frac{BK^{2}}{K^{2}-A}\left[\frac{\left(1+LK\tanh K\right)\cosh\left(\sqrt{A}\bar{z}\right)}{\cosh\sqrt{A}+L\sqrt{A}\sinh\sqrt{A}}-\frac{\cosh\left(K\bar{z}\right)}{\cosh\left(K\right)}\right]. \end{array} \end{array}\right.$$

The velocity can be expressed by inverse of a Fourier transform as follows:

(21) $$\left\{\begin{array}{c} \begin{array}{c} \bar{u}=\frac{1}{2\pi }\int_{-\infty }^{+\infty }\frac{BH\;aG}{A}\left[1-\frac{\cosh\left(\sqrt{A}\bar{z}\right)}{\cosh\sqrt{A}+L\sqrt{A}\sinh\sqrt{A}}\right]e^{s\bar{t}}\mathrm{ds},\\ \bar{v}=\frac{1}{2\pi }\int_{-\infty }^{+\infty }\frac{BK^{2}}{K^{2}-A}\left[\frac{\left(1+LK\tanh K\right)\cosh\left(\sqrt{A}\bar{z}\right)}{\cosh\sqrt{A}+L\sqrt{A}\sinh\sqrt{A}}-\frac{\cosh\left(K\bar{z}\right)}{\cosh\left(K\right)}\right]e^{s\bar{t}}\mathrm{ds}. \end{array} \end{array}\right.$$

For a given external electric field, the velocity can be obtained from Equation (21).

### 2.4. Numerical Solutions of the EMHD Velocity Field

The numerical solutions can also be provided through the finite difference method. First, a uniform discretization is introduced over the rectangular computational domain $\left(D=\{\left(\bar{z},\bar{t}\right)|\bar{z},\bar{t}\in \left[-1,1\right]\times \left[0,T\right]\}\right)$ by ${\bar{z}}_{j}=j\Delta z$, j=0,1,2,⋯,M; ${\bar{t}}_{n}=n\Delta t$, n=0,1,2,⋯,N, in which Δz=2/M, and Δt=T/N are the spatial and temporal step sizes, respectively. For given $g(\bar{z},\bar{t})$, the following approximations are introduced

(22) $$\frac{\partial g(\bar{z},\bar{t})}{\partial \bar{t}}{|}_{\bar{z}={\bar{z}}_{j}}^{\bar{t}={\bar{t}}_{n}}\approx \frac{g_{j+1}^{n}-g_{j}^{n}}{\Delta t},$$

(23) $$\frac{\partial^{2}g\left(\bar{z},\bar{t}\right)}{\partial {\bar{z}}^{2}}{|}_{\bar{z}={\bar{z}}_{j}}^{\bar{t}={\bar{t}}_{n}}\approx \frac{g_{j+1}^{n}-2g_{j}^{n}+g_{j-1}^{n}}{\Delta z^{2}}.$$

Based on the $L_{1}$ and $L_{2}$ approximations of the Caputo fractional derivative [27], the following approximations are obtained:

(24) $$D_{\bar{t}}^{\gamma }g\left(\bar{z},\bar{t}\right){|}_{\bar{z}={\bar{z}}_{j}}^{\bar{t}={\bar{t}}_{n}}=\frac{\Delta t^{-\gamma }}{\Gamma (2-\gamma )}\sum_{j=0}^{n-1}b_{j}^{\left(\gamma \right)}\left(g_{{\bar{z}}_{j}}^{n-j}-g_{{\bar{z}}_{j}}^{n-j-1}\right),\;0\leq \gamma <1,$$

(25) $$D_{\bar{t}}^{\gamma }g\left(\bar{z},\bar{t}\right){|}_{\bar{z}={\bar{z}}_{j}}^{\bar{t}={\bar{t}}_{n}}=\frac{\Delta t^{-\gamma }}{\Gamma (3-\gamma )}\sum_{j=0}^{n-1}b_{j}^{\left(\gamma -1\right)}\left(g_{{\bar{z}}_{j}}^{n-j+1}-2g_{{\bar{z}}_{j}}^{n-j}+g_{{\bar{z}}_{j}}^{n-j-1}\right),\;1\leq \gamma <2,$$

(26) $$D_{\bar{t}}^{-\gamma }g\left(\bar{z},\bar{t}\right){|}_{\bar{z}={\bar{z}}_{j}}^{\bar{t}={\bar{t}}_{n}}=\frac{\Delta t^{-\gamma }}{2\Gamma (1-\gamma )}\sum_{j=0}^{n-1}b_{j}^{\left(1+\gamma \right)}\left(g_{{\bar{z}}_{j}}^{n-j}+g_{{\bar{z}}_{j}}^{n-j-1}\right),\;\gamma >0,$$

where $b_{j}^{\left(\gamma \right)}={\left(j+1\right)}^{1-\gamma }-j^{1-\gamma }$$\left(b_{0}^{\left(\gamma \right)}=1\right)$.

The discretized finite difference scheme of Equation (14) is as follows:

(27) $$\begin{array}{cc} \; & \frac{De^{\alpha }\Delta t^{-\alpha -1}}{\Gamma (2-\alpha )}\sum_{i=0}^{n-1}b_{i}^{\left(\alpha \right)}\left({\bar{u}}_{j}^{n-i+1}-2{\bar{u}}_{j}^{n-i}+{\bar{u}}_{j}^{n-i-1}\right)+\frac{{\bar{u}}_{j}^{n+1}-{\bar{u}}_{j}^{n}}{\Delta t}\\ \; & +\frac{H\;a^{2}De^{\alpha }\Delta t^{-\alpha }}{\Gamma (2-\alpha )}\sum_{i=0}^{n-1}b_{i}^{\left(\alpha \right)}\left({\bar{u}}_{j}^{n-i+1}-{\bar{u}}_{j}^{n-i}\right)+H\;a^{2}{\bar{u}}_{j}^{n}\\ \; & =\frac{De^{\beta -1}\Delta t^{1-\beta }}{\Gamma (2-\beta )}\sum_{i=0}^{n-1}b_{i}^{\left(\beta \right)}\frac{{\bar{u}}_{j+1}^{n-i}-2{\bar{u}}_{j}^{n-i}+{\bar{u}}_{j-1}^{n-i}}{\Delta z^{2}}\\ \; & +\frac{H\;aGDe^{\alpha }\Delta t^{-\alpha }}{\Gamma (2-\alpha )}\sum_{i=0}^{n-1}b_{i}^{\left(\alpha \right)}\left[\bar{E}\left({\bar{t}}_{n-i}\right)-\bar{E}\left({\bar{t}}_{n-i-1}\right)\right]+H\;aG\bar{E}\left({\bar{t}}_{n}\right),\\ \; & \;n=0,1,2,\cdots ,N-1,\;j=1,2,3,\cdots ,M-1, \end{array}$$

(28) $$\begin{array}{cc} \; & \frac{De^{\alpha }\Delta t^{-\alpha -1}}{\Gamma (2-\alpha )}\sum_{i=0}^{n-1}b_{i}^{\left(\alpha \right)}\left({\bar{v}}_{j}^{n-i+1}-2{\bar{v}}_{j}^{n-i}+{\bar{v}}_{j}^{n-i-1}\right)+\frac{{\bar{v}}_{j}^{n+1}-{\bar{v}}_{j}^{n}}{\Delta t}\\ \; & +\frac{H\;a^{2}De^{\alpha }\Delta t^{-\alpha }}{\Gamma (2-\alpha )}\sum_{i=0}^{n-1}b_{i}^{\left(\alpha \right)}\left({\bar{v}}_{j}^{n-i+1}-{\bar{v}}_{j}^{n-i}\right)+H\;a^{2}{\bar{v}}_{j}^{n}\\ \; & =\frac{De^{\beta -1}\Delta t^{1-\beta }}{\Gamma (2-\beta )}\sum_{i=0}^{n-1}b_{i}^{\left(\beta \right)}\frac{{\bar{v}}_{j+1}^{n-i}-2{\bar{v}}_{j}^{n-i}+{\bar{v}}_{j-1}^{n-i}}{\Delta z^{2}}\\ \; & +\frac{K^{2}De^{\alpha }\Delta t^{-\alpha }}{\Gamma (2-\alpha )}\frac{\cosh\left(K{\bar{z}}_{j}\right)}{\cosh K}\sum_{i=0}^{n-1}b_{i}^{\left(\alpha \right)}\left[\bar{E}\left({\bar{t}}_{n-i}\right)-\bar{E}\left({\bar{t}}_{n-i-1}\right)\right]+K^{2}\frac{\cosh\left(K{\bar{z}}_{j}\right)}{\cosh K}\bar{E}\left({\bar{t}}_{n}\right),\\ \; & \;n=0,1,2,\cdots ,N-1,\;j=1,2,3,\cdots ,M-1. \end{array}$$

Here, Equations (27) and (28) are the discrete schemes of the *x*-axis and *y*-axis directions, respectively.

The discretized initial conditions are

(29) $$\left\{\begin{array}{c} \begin{array}{c} {\bar{u}}_{j}^{0}=\bar{u}\left(0\right),\;\frac{{\bar{u}}_{j}^{1}-{\bar{u}}_{j}^{0}}{\Delta t}={\bar{u}}^{\prime }\left(0\right),\;j=0,1,2,\cdots ,M,\\ {\bar{v}}_{j}^{0}=\bar{v}\left(0\right),\;\frac{{\bar{v}}_{j}^{1}-{\bar{v}}_{j}^{0}}{\Delta t}={\bar{v}}^{\prime }\left(0\right),\;j=0,1,2,\cdots ,M, \end{array} \end{array}\right.$$

where $\bar{u}\left(0\right)$ and $\bar{v}\left(0\right)$ are the values of Equation (21) at $\bar{t}=0$, ${\bar{u}}^{\prime }\left(0\right)$ and ${\bar{v}}^{\prime }\left(0\right)$ are the values at $\bar{t}=0$ after the derivative of Equation (21) with respect to $\bar{t}$. The discretized boundary conditions are

(30) $$\left\{\begin{array}{c} \begin{array}{c} {\bar{u}}_{0}^{n}-L\frac{{\bar{u}}_{1}^{n}-{\bar{u}}_{0}^{n}}{\Delta z}=0,\;{\bar{u}}_{M}^{n}+L\frac{{\bar{u}}_{M-1}^{n}-{\bar{u}}_{M}^{n}}{\Delta z}=0,\;n=0,1,2,\cdots ,N,\\ {\bar{v}}_{0}^{n}-L\frac{{\bar{v}}_{1}^{n}-{\bar{v}}_{0}^{n}}{\Delta z}=0,\;{\bar{v}}_{M}^{n}+L\frac{{\bar{v}}_{M-1}^{n}-{\bar{v}}_{M}^{n}}{\Delta z}=0,\;n=0,1,2,\cdots ,N. \end{array} \end{array}\right.$$

## 3. Results and Discussion

In the previous section, through the method of Fourier transform, the analytical expression of the velocity was derived for the EMHD flow of fractional Maxwell fluids between micro-parallel plates. There are three dimensionless numbers involved in Equation (13), namely, Hartmann number Ha, dimensionless parameter *G* and Deborah number De. The Hartmann number gives an estimate of the magnetic forces compared to the viscous forces, and *G* is a non-dimensional parameter representing the strength of the *x* direction electric field [44]. The Deborah number De is a dimensionless quantity in rheology, which is used to describe the fluidity of materials under certain conditions. It can be used as one of the parameters to measure the viscoelasticity of the fluid. It is defined as the ratio between the relaxation time and the observation time of the mechanical response of the material under the observation conditions. A definition of the Deborah number is given in the research of Ding [19,49], and this definition is also adopted in this paper to study the resonance behavior in the flow process.

**Figure 2:**
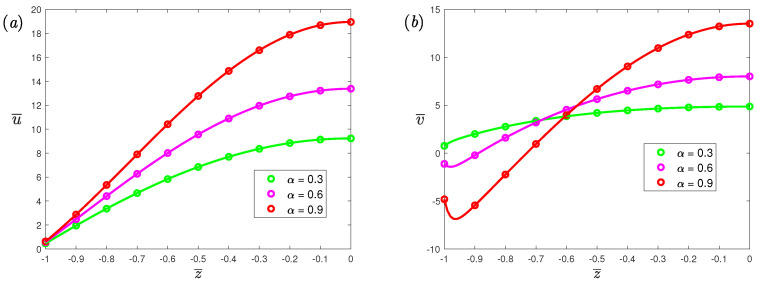
The dimensionless velocity profiles, (**a**) spanwise; (**b**) streamwise, of fractional Maxwell model across half channel width for different dimensionless parameters α at $\bar{t}=\pi /3$, De=5, β=0.9, L=0.03, K=50, $\bar{\omega }=1$, Ha=0.5, G=20, where the symbol represents numerical solutions and the curve represent the analytical solutions.

In this section, an applied periodic electric field $E=E_{0}\cos\left(\omega t\right)$ with the dimensionless frequency $\bar{\omega }=\rho H^{2}\omega /\mu$ is considered. The ‘ifourier’ command in Matlab symbolic calculation is used to obtain the analytical solution of the velocity. The comparison between the numerical and the analytical solutions is shown in the Figure 2, which shows that they have a good agreement. The fractional derivative parameters α and β also play a significant role in the flow, where 0≤α≤β≤1. Friedrich [50] proved that the rheological constitutive equation exhibits fluid-like behavior only in the case where β=1, so the fractional Maxwell fluid model with single fractional parameter α is considered. Based on Ref. [44], the electrical conductivity is σ∼$2.2\times 10^{-4}$–4$\times 10^{3}\;S/m$, viscosity is μ∼$10^{-3}$–1.5$\times 10^{-3}\;\mathrm{kg}/\left(\mathrm{ms}\right)$, the imposed electrical field is $E_{0}$∼0–30 V/m, the imposed magnetic field is *B*∼0.01–5 T, and the Hartmann number is from 0 to 10. Thus, the order of *G* can be evaluated, and its range is changed from 0 to $6\times 10^{3}$; here, it is fixed as 20. This means that the physical configuration can be uniquely determined given the values of these parameters that satisfy the above conditions. For example, when $U_{eo}$∼$2\times 10^{-4}$, σ∼$4\times 10^{2}$, μ∼$1\times 10^{2}$, $E_{0}$∼20V/m, *h*∼$1\times 10^{-4}$, it corresponds to a physical study of the influence of magnetic field strength and fluid elasticity on the flow.

### 3.1. Velocity Distribution

**Figure 3:**
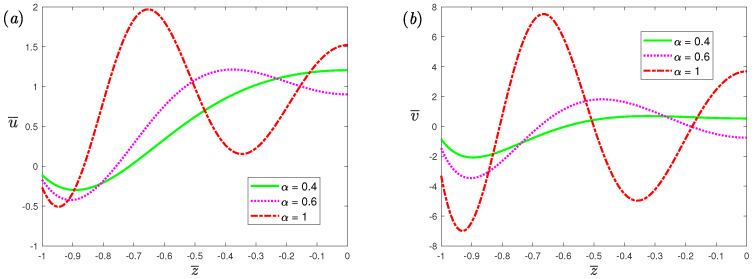
The dimensionless velocity profiles, (**a**) spanwise; (**b**) streamwise, of fractional Maxwell model across half channel width for different dimensionless parameters α at $\bar{t}=\pi /6$, β=1, De=5, L=0.03, K=10, $\bar{\omega }=5$, Ha=0.5.

**Figure 4:**
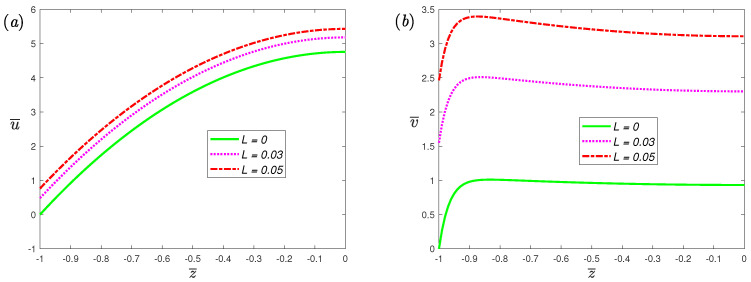
The dimensionless velocity, (**a**) spanwise; (**b**) streamwise, profiles of fractional Maxwell model across half channel width for different dimensionless parameters *L* at α=0.7, β=1, De=5, K=30, $\bar{\omega }=0$, Ha=0.5.

Velocity profiles for different fractional parameters α at Hartmann number Ha=0.5 are shown in Figure 3. From Figure 3, it can be observed that the amplitude of velocity increases as the α gradually increases. An increased α increases the slip velocity at the wall. From Equation (15), the slip velocity is the product of the fluid shear rate at the wall and the slip length. Therefore, increasing the value of α will increase the fluid shear rate at the wall since the slip length is fixed, thereby promoting slip velocity. Figure 4 exhibits the velocity profiles for different dimensionless slip lengths at dimensionless frequency $\bar{\omega }=0$. It can be seen that as the dimensionless slip length increases, the slip velocity at the wall increases. In addition, when *L* increases, we can see that the velocity amplitude also has the same elevated trend as the slip velocity. From Figure 4b, the concave part in the middle is caused by the Lorentz force, opposite to the electro-osmotic force.

**Figure 5:**
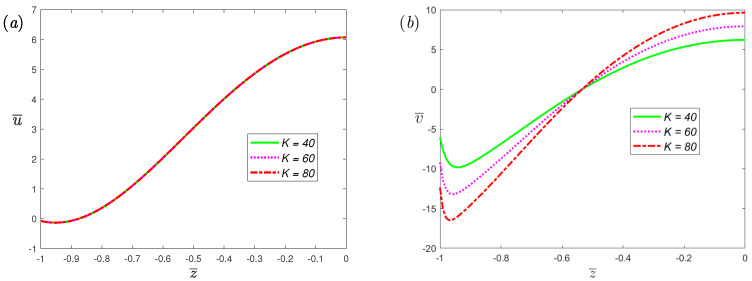
The dimensionless velocity, (**a**) spanwise; (**b**) streamwise, profiles of fractional Maxwell model across half channel width for different dimensionless parameters *K* at $\bar{t}=\pi /3$, α=0.7, β=1, De=5, L=0.03, $\bar{\omega }=2$, Ha=0.5.

**Figure 6:**
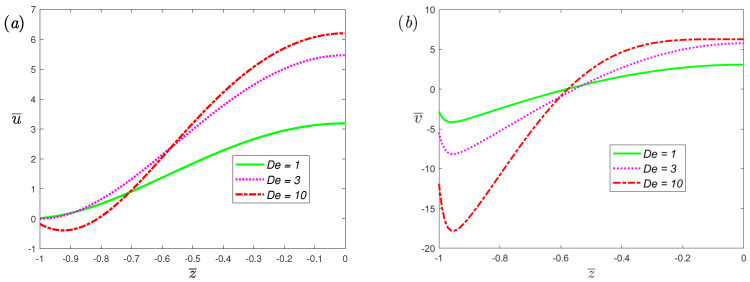
The dimensionless velocity, (**a**) spanwise; (**b**) streamwise, profiles of fractional Maxwell model across half channel width for different dimensionless parameters De at $\bar{t}=\pi /3$, α=0.7, β=1, K=50, L=0.03, $\bar{\omega }=2$, Ha=0.5.

The variations of velocity profiles with half channel width at different values of the electrokinetic width *K* for α=0.7 and β=1 are plotted in Figure 5. There is no change in the velocity in the *x*-axis, which means that the flow perpendicular to the direction of the electric and magnetic fields is induced by the Lorentz force and has nothing to do with the electric double layer; this can be found from Equation (9). In the direction of flow induced by electro-osmotic force, both the slip velocity and amplitude of velocity will increase with the increase in *K*. Figure 6 shows the effects of the dimensionless Deborah number De on the velocity profiles of EMHD flow for fractional Maxwell fluids. From the figure, an increase in De makes the slip velocity and amplitude of velocity increase. In a physical sense, the relaxation time (its magnitude is characterized by De) refers to the time required for a viscoelastic fluid to return to its normal state after deformation. Its magnitude can reflect the elasticity of the fluid. The elastic effect of the fluid can enhance the flow, which has been verified by experiments [51].

**Figure 7:**
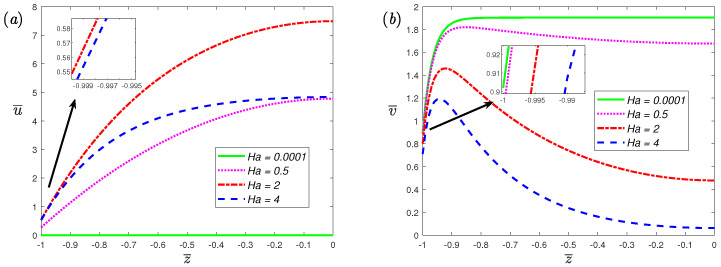
The absolute value of dimensionless velocity, (**a**) spanwise; (**b**) streamwise, for fractional Maxwell model across half channel width for different dimensionless parameters Ha at α=0.7, β=1, K=30, L=0.03, De=5, $\bar{\omega }=0$.

Figure 7 illustrates the relationship between velocity profiles and Hartmann number Ha for α=0.7 and β=1. In order to simplify the discussion, the steady case ($\bar{\omega }=0$) is considered, and for this case, u>0, v>0. In the direction perpendicular to the electric and magnetic fields, the critical value $H\;a_{c}≃2$ can be found in the figure. When $H\;a<H\;a_{c}$, the wall slip velocity and velocity amplitude increase with the increase in Ha, and when $H\;a>H\;a_{c}$, they decrease with the increase in Ha. Along the direction of the electric field, the slip velocity and the amplitude of the velocity will decrease with increasing Ha. Moreover, at the center of the microchannel, it can be found that a larger value of Ha will have a strong inhibitory effect on the velocity. As Ha increases, the velocity at the center gradually tends to 0. This result can be explained by the force on the fluid in the microchannel. From Equation (9), the body force of the fluid in the *x*-axis direction and the *y*-axis direction are $\sigma B\left(E-Bu\right)$ and $\rho_{e}E-\sigma B^{2}v$, respectively, and the dimensionless forms are $H\;aG-H\;a^{2}\bar{u}$ and $K^{2}\;\cosh\left(K\bar{z}\right)/\cosh\left(K\right)-H\;a^{2}\bar{v}$. In the *x*-axis direction, the body force on the fluid first increases with the increase in Ha, reaches the maximum value at $G/\left(2\bar{u}\right)≃H\;a_{c}$, and then decreases with the increase in Ha. Along the direction of the electric field, the body force on the fluid always decreases as Ha increases. Increasing the external force acting on the fluid will increase the velocity amplitude and slip velocity at the wall.

### 3.2. Resonance Behavior of Fractional Viscoelastic Fluids

In this part, we discuss the flow behavior of fractional viscoelastic fluids through the volumetric flow rate of the fluid. The dimensionless volumetric flow rate is defined as

(31) $$\bar{Q}=\int_{-1}^{1}U\left(\bar{z},\bar{t}\right)d\;\bar{z}.$$

The variations of the volumetric flow rate for fractional Maxwell model are given graphically at four parameters (fractional parameter α, dimensionless slip length *L*, Deborah number De, and Hartmann number Ha) in detail. It can be seen from these figures that drastic enhancements of the volumetric flow rate occur at certain frequencies, which reflect resonance phenomena. When resonance occurs, it is obvious that the amplitude (peak) of the enhancement decreases as the frequency increases, and the largest enhancement occurs at the smallest resonance frequency. In addition, in the *x*-axis direction, the resonance peak at the minimum frequency is large, and then the peaks drops sharply. Therefore, in the x-axis direction, the resonance decay is fast.

**Figure 8:**
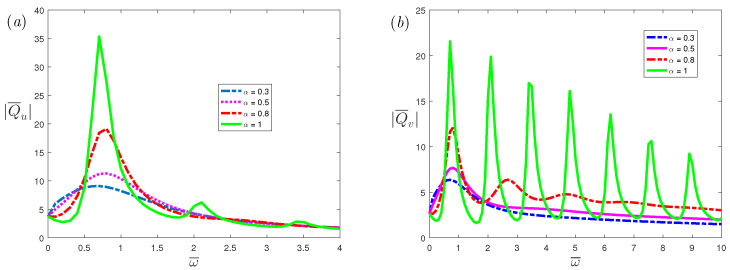
The dimensionless mean volumetricflow rate, (**a**) spanwise; (**b**) streamwise, of fractional Maxwell model with the dimensionless frequency $\bar{\omega }$ for different fractional parameters α at β=1, K=20, L=0.03, De=5, Ha=0.3.

**Figure 9:**
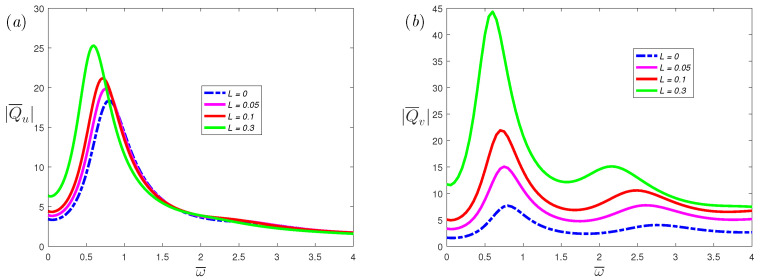
The dimensionless mean volumetric flow rate, (**a**) spanwise; (**b**) streamwise, of fractional Maxwell model with the dimensionless frequency $\bar{\omega }$ for different dimensionless parameters *L* at α=0.8, β=1, K=20, De=5, Ha=0.3.

**Figure 10:**
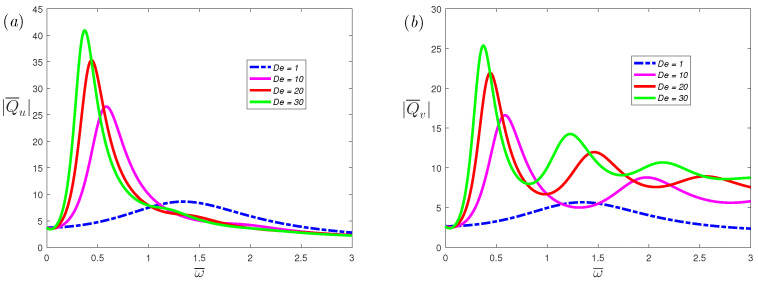
The dimensionless mean volumetric flow rate, (**a**) spanwise; (**b**) streamwise, of fractional Maxwell model with the dimensionless frequency $\bar{\omega }$ for different dimensionless parameters De at α=0.8, β=1, K=20, L=0.03, Ha=0.3.

Figure 8 illustrates the effects of fractional parameters α on the volumetric flow rate of the *x*-axis and *y*-axis, respectively. From the figure, it can be observed that as the α increases, the resonance peak increases. When α decreases, the number of resonance peaks will decrease. This indicates that the resonance behavior of the fractional viscoelastic fluid is suppressed compared with the classical Maxwell fluid. Figure 9 and Figure 10 depicts the volumetric flow rate of the *x*-axis and *y*-axis with $\bar{\omega }$. It is noticed that the increase in slip length *L* or Deborah number De can not only enhance resonance, but also reduce the frequency at which resonance occurs.

**Figure 11:**
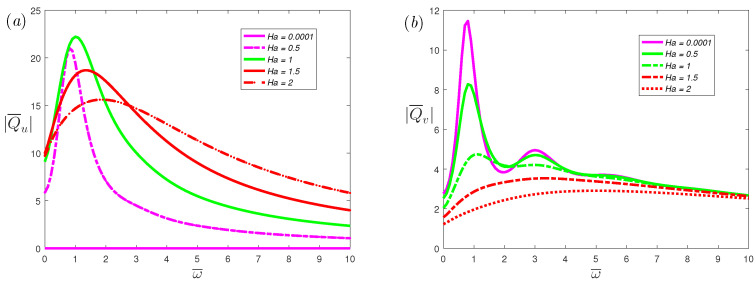
The dimensionless mean volumetric flow rate, (**a**) spanwise; (**b**) streamwise, of the fractional Maxwell model with the dimensionless frequency $\bar{\omega }$ for different dimensionless parameters Ha at α=0.7, β=1, K=20, De=5, L=0.03.

Figure 11 shows the effects of Hartmann number Ha on volumetric flow rate of the *x*-axis and *y*-axis, respectively. From Figure 11a, a very small Hartmann number will result in a very small volumetric flow rate. When the Hartmann number increases, the volumetric flow rate and resonance peak will increase first, and then decrease. The volumetric flow rate reaches the maximum when Ha≃1. The volumetric flow rate represents the net flow of the fluid in the flow process. A slight change in some positions will not affect the value of Ha, which can maximize the volumetric flow rate. However, in Section 3.1, the critical number $H\;a_{c}$ is the value that makes all positions reach the maximum velocity. Therefore, Ha that makes the volumetric flow rate reach the maximum is different from the critical number $H\;a_{c}$. From Figure 11b, the peaks of the resonance will decrease as Ha increases. It should be mentioned that a larger Ha always suppresses resonance. This means that when a suitable external magnetic field is applied, the resonance behavior will be eliminated.

## 4. Conclusions

The analytical solutions of combined unsteady electro-osmotic and magnetohydrodynamic velocity are obtained through using the Fourier transform method for the fractional Maxwell model in a slit parallel plate microchannel. Similarly, a numerical solution can also be provided by the finite difference method. The velocity and resonance behavior of periodic EMHD flow are shown graphically and discussed in detail. The resonance greatly enhances the relevant quantities, and the appearance of resonance behavior depends on the value of α,L,De,Ha. The main conclusions are as follows: (i) The fractional derivative parameter α, the dimensionless slip length *L*, and the Deborah number De promote the velocity amplitude and slip velocity, and enhance the resonance behavior. (ii) The electrokinetic width *K* promotes the flow along the direction of the electric field, and the change of *K* does not affect the flow in the direction perpendicular to the electric and magnetic fields. (iii) In the direction perpendicular to the electric and magnetic fields, when Ha increases, the flow will be promoted first and then suppressed; in the direction of the electric field, Ha has an inhibitory effect on the flow.

## Data Availability

Not applicable.

## References

[1] Buren M., Jian Y., Chang L., Li F., Liu Q. (2017). Combined electromagnetohydrodynamic flow in a microparallel channel with slightly corrugated walls. Fluid Dyn. Res..

[2] Harnett C.K., Templeton J., Dunphy-Guzman K.A., Senousy Y.M., Kanouff M.P. (2008). Model based design of a microfluidic mixer driven by induced charge electroosmosis. Lab Chip.

[3] Ding Z., Jian Y., Tan W. (2019). Electrokinetic energy conversion of two-layer fluids through nanofluidic channels. J. Fluid Mech..

[4] Cruz D., Pinho F. (2007). Fully-developed pipe and planar flows of multimode viscoelastic fluids. J. Non-Newtonian Fluid Mech..

[5] Sadek S.H., Pimenta F., Pinho F.T., Alves M.A. (2017). Measurement of electroosmotic and electrophoretic velocities using pulsed and sinusoidal electric fields. Electrophoresis.

[6] Sadr R., Yoda M., Zheng Z., Conlisk A. (2004). An experimental study of electro-osmotic flow in rectangular microchannels. J. Fluid Mech..

[7] Kofler M., Lenninger M., Mayer G., Neuwirt H., Grimm M., Bechtold T. (2016). Multi-chamber electroosmosis using textile reinforced agar membranes—A promising concept for the future of hemodialysis. Carbohydr. Polym..

[8] Wang X., Xu H., Qi H. (2020). Numerical analysis for rotating electro-osmotic flow of fractional Maxwell fluids. Appl. Math. Lett..

[9] Vinita, Poply V., Devi R. (2021). A two-component modeling for free stream velocity in magnetohydrodynamic nanofluid flow with radiation and chemical reaction over a stretching cylinder. Heat Transfer.

[10] Buren M., Jian Y., Chang L., Liu Q., Zhao G. (2017). AC magnetohydrodynamic slip flow in microchannel with sinusoidal roughness. Microsyst. Technol..

[11] Jian Y., Si D., Chang L., Liu Q. (2015). Transient rotating electromagnetohydrodynamic micropumps between two infinite microparallel plates. Chem. Eng. Sci..

[12] Si D., Jian Y. (2015). Electromagnetohydrodynamic (EMHD) micropump of Jeffrey fluids through two parallel microchannels with corrugated walls. J. Phys. D Appl. Phys..

[13] Seth G., Mandal P. (2019). Analysis of electromagnetohydrodynamic stagnation point flow of nanofluid over a nonlinear stretching sheet with variable thickness. J. Mech..

[14] Buren M., Jian Y. (2015). Electromagnetohydrodynamic (EMHD) flow between two transversely wavy microparallel plates. Electrophoresis.

[15] Khan M.I., Haq F., Khan S.A., Hayat T., Khan M.I. (2019). Development of thixotropic nanomaterial in fluid flow with gyrotactic microorganisms, activation energy, mixed convection. Comput. Methods Programs Biomed..

[16] Khan M.I., Alsaedi A., Qayyum S., Hayat T., Khan M.I. (2019). Entropy generation optimization in flow of Prandtl-Eyring nanofluid with binary chemical reaction and Arrhenius activation energy. Colloids Surf..

[17] Ivanov A. (2020). Distribution Features of Electromagnetic and Hydrodynamic Fields in the Conductive Electric-Current Treatment of Melts Using Parallel Electrodes. Surf. Eng. Appl. Electrochem..

[18] Chakraborty S., Paul D. (2006). Microchannel flow control through a combined electromagnetohydrodynamic transport. J. Phys. D Appl. Phys..

[19] Ding Z., Jian Y. (2021). Resonance behaviors in periodic viscoelastic electrokinetic flows: A universal Deborah number. Phys. Fluids.

[20] Letelier M.F., Siginer D.A., Almendra D.L., Stockle J.S. (2019). Resonance in laminar pipe flow of non-linear viscoelastic fluids. Int. J. Non-Linear Mech..

[21] Yin Y., Zhu K. (2006). Oscillating flow of a viscoelastic fluid in a pipe with the fractional Maxwell model. Appl. Math. Comput..

[22] Andrienko Y.A., Siginer D.A., Yanovsky Y.G. (2000). Resonance behavior of viscoelastic fluids in Poiseuille flow and application to flow enhancement. Int. J. Non-Linear Mech..

[23] Lambert A.A., Cuevas S., del Rio J.A., López de Haro M. (2009). Heat transfer enhancement in oscillatory flows of Newtonian and viscoelastic fluids. Int. J. Heat Mass Transfer.

[24] Tsiklauri D., Beresnev I. (2001). Enhacement in the dymanic response of a viscoelastic fluid flowing through a longitudinally vibrating tube. Phys. Rev. E.

[25] Emilio Herrera E., Calderas F., Chávez A., Manero O. (2010). Study on the pulsating flow of a worm-like micellar solution. J. Non-Newtonian Fluid Mech..

[26] Su X., Chen W., Xu W. (2017). Characterizing the rheological behaviors of non-Newtonian fluid via a viscoelastic component: Fractal dashpot. Adv. Mech. Eng..

[27] Guo B., Pu X., Huang F. (2015). Fractional Partial Differential Equations and Their Numerical Solutions.

[28] Tripathi D. (2011). Peristaltic transport of fractional Maxwell fluids in uniform tubes: Applications in endoscopy. Comput. Math. with Appl..

[29] Feng C., Si X., Cao L., Zhu B. (2021). The slip flow of generalized Maxwell fluids with time-distributed characteristics in a rotating microchannel. Appl. Math. Lett..

[30] Cao L., Zhang P., Li B., Zhu J., Si X. (2021). Numerical study of rotating electro-osmotic flow of double layers with a layer of fractional second-order fluid in a microchannel. Appl. Math. Lett..

[31] Yang X., Qi H., Jiang X. (2018). Numerical analysis for electroosmotic flow of fractional Maxwell fluids. Appl. Math. Lett..

[32] Abdulhameed M., Vieru D., Roslan R. (2017). Magnetohydrodynamic electroosmotic flow of Maxwell fluids with Caputo—Fabrizio derivatives through circular tubes. Comput. Math. Appl..

[33] Abro K.A., Solangi M.A. (2017). Heat transfer in magnetohydrodynamic second grade fluid with porous impacts using Caputo—Fabrizoi fractional derivatives. Punjab Univ. J. Math..

[34] Liu Y., Zhang H., Jiang X. (2021). Fast evaluation for magnetohydrodynamic flow and heat transfer of fractional Oldroyd-B fluids between parallel plates. Z. Angew. Math. Mech..

[35] Baudry J., Charlaix E., Tonck A., Mazuyer D. (2001). Experimental Evidence for a Large Slip Effect at a Nonwetting Fluid-Solid Interface. Langmuir.

[36] Bonaccurso E., Kappl M., Butt H.J. (2002). Hydrodynamic Force Measurements: Boundary Slip of Water on Hydrophilic Surfaces and Electrokinetic Effects. Phys. Rev. Lett..

[37] Snoeijer J.H., Delon G., Fermigier M., Andreotti B. (2006). Avoided Critical Behavior in Dynamically Forced Wetting. Phys. Rev. Lett..

[38] Bonaccurso E., Butt H.J., Craig V.S. (2003). Surface roughness and hydrodynamic boundary slip of a Newtonian fluid in a completely wetting system. Phys. Rev. Lett..

[39] Craig V.S.J., Neto C., Williams D.R.M. (2001). Shear-dependent boundary slip in an aqueous Newtonian liquid. Phys. Rev. Lett..

[40] Pascall A.J., Squires T.M. (2010). Induced Charge Electro-osmosis over Controllably Contaminated Electrodes. Phys. Rev. Lett..

[41] Galea T.M., Attard P. (2004). Molecular dynamics study of the effect of atomic roughness on the slip length at the fluid-solid boundary during shear flow. Langmuir.

[42] Bouzigues C.I., Tabeling P., Bocquet L. (2008). Nanofluidics in the Debye Layer at Hydrophilic and Hydrophobic Surfaces. Phys. Rev. Lett..

[43] Khair A.S., Squires T.M. (2009). The influence of hydrodynamic slip on the electrophoretic mobility of a spherical colloidal particle. Phys. Fluids.

[44] Jian Y. (2015). Transient MHD heat transfer and entropy generation in a microparallel channel combined with pressure and electroosmotic effects. Int. J. Heat Mass Transf..

[45] Yang J., Kwok D.Y. (2003). Microfluid Flow in Circular Microchannel with Electrokinetic Effect and Navier’s Slip Condition. Langmuir.

[46] Xu M., Jian Y. (2020). Unsteady rotating electroosmotic flow with time-fractional Caputo—Fabrizio derivative. Appl. Math. Lett..

[47] Tan W., Pan W., Xu M. (2003). A note on unsteady flows of a viscoelastic fluid with the fractional Maxwell model between two parallel plates. Int. J. Non Linear Mech..

[48] Namias V. (1980). The Fractional Order Fourier Transform and its Application to Quantum Mechanics. Geoderma.

[49] Ding Z., Jian Y. (2021). Electrokinetic oscillatory flow and energy conversion of viscoelastic fluids in microchannels: A linear analysis. J. Fluid Mech..

[50] Friedrich C. (1991). Relaxation and retardation functions of the Maxwell model with fractional derivatives. Rheol. Acta.

[51] Casanellas L., Ortín J. (2012). Experiments on the laminar oscillatory flow of wormlike micellar solutions. Rheol. Acta.

